# Electrospun Nanostructured Fibers of Collagen-Biomimetic Apatite on Titanium Alloy

**DOI:** 10.1155/2012/123953

**Published:** 2012-02-08

**Authors:** Michele Iafisco, Ismaela Foltran, Simona Sabbatini, Giorgio Tosi, Norberto Roveri

**Affiliations:** ^1^Dipartimento di Chimica “G. Ciamician,” Alma Mater Studiorum Università di Bologna, Via Selmi 2, 40126 Bologna, Italy; ^2^Dipartimento di Scienze Mediche, Università del Piemonte Orientale, Via Solaroli 17, 28100 Novara, Italy; ^3^Dipartimento di Scienze e Tecnologie Chimiche, Università Politecnica delle Marche, Via Brecce Bianche, 60131 Ancona, Italy

## Abstract

Titanium and its alloys are currently the mainly used materials to manufacture orthopaedic implants due to their excellent mechanical properties and corrosion resistance. Although these materials are bioinert, the improvement of biological properties (e.g., bone implant contact) can be obtained by the application of a material that mimics the bone extracellular matrix. To this aim, this work describes a new method to produce nanostructured collagen-apatite composites on titanium alloy substrate, by combining electrospinning and biomimetic mineralization. The characterization results showed that the obtained mineralized scaffolds have morphological, structural, and chemical compositional features similar to natural bone extracellular matrix. Finally, the topographic distribution of the chemical composition in the mineralized matrix evaluated by Fourier Transform Infrared microspectroscopy demonstrated that the apatite nanocrystals cover the collagen fibers assembled by the electrospinning.

## 1. Introduction

Titanium and its alloy are currently the mainly used materials for biomedical and dental implants for their good mechanical properties, high corrosion resistance, and excellent biocompatibility [1]. However, these materials are bioinert since they can nonspecifically downregulate biological responses. For this reason, the research has been directed to the surface modification of these materials to improve bone implant contact and their biological properties. Among the different materials used for the surface modification of Ti substrates, the best way could be the application of a coating that replaces the extracellular matrix (ECM) of bone, basically constituted of biomimetic apatite nanocrystals (HA) and collagen fibers [2].

The mineral phase of bone and teeth consists of plate-like shape nonstoichiometric carbonated-HA crystals with length of about 100 nm, width of 20–30 nm, thickness of 3–6 nm, and different types, and foreign ions substitutions including sodium, magnesium, potassium, carbonate, and fluoride [3, 4]. There are several methods to synthesise hydroxyapatite but mainly it is possible to distinguish two types of hydroxyapatite based on the temperature of synthesis: a high-temperature sintered form (>900°C) and a low-temperature precipitate (~37°C). The compounds synthesized at low-temperature exhibit more solubility, much greater surface area, and they are inherently more active in biological systems in contrast to the biologically inert sintered versions [5]. Sintered hydroxyapatite is most commonly used in load-bearing orthopaedic implants, on the other hand it is not bioresorbable as they are stable over the lifetime of the individual. Since the bone mineralization process occurred at basic pH and physiological temperature (37°C), the synthetic procedure that uses these parameters is called biomimetic mineralization. The final HA formed, in spite of having variable composition, is predominantly carbonated apatite with low crystallinity and it is very similar to the form found in bone, a favourable factor from a tissue engineering perspective [6].

Recent studies concerning collagen coatings on Ti implants have demonstrated their effective role in stimulating cellular responses, increasing bone remodelling, and improving bone growth and bone implant contact [7, 8]. Therefore, hybrids of collagen/HA have greater potential for clinical applications than the pure HA or collagen equivalents, because they benefit from the advantageous properties of both materials. In the composite, the collagen component can act as a matrix to embed the HA particles, alleviating the brittleness of HA and promoting the adhesion of the particles to Ti substrate [9]. At the same time, the HA used as a filler of the protein could improve the mechanical strength and biological performance of the collagen.

Electrospinning process has attracted a great deal of attention in the last years as a way to reproduce the structure of natural ECM by means of producing fibers in the nanometre scale [10]. This technique is used to fabricate nanofibrous structures from natural and synthetic polymers such as collagen, chitosan, silk fibroin, poly(lactide), polyurethane, polycaprolactone, and so forth [11–14]. Several papers reported the production of nanofibrous materials made of collagen mixed with biocompatible synthetic polymers using the electrospinning technique, as well as HA-polymeric fibers [15–18]. Most of these studies have demonstrated enhanced bioactivity of the composite materials, especially in nanofibrous constructs [19, 20]. However, the data on the production of pure nano-structured collagen fibers and HA-collagen composites by electrospining, as well as the data on the deposition of them on titanium alloy or on other orthopaedic implants, are extremely scarce or have not been investigated up to now.

The aim of this study was to develop a method to produce nanostructured collagen/HA composite with characteristics similar to natural-bone ECM on titanium alloy substrates, by combining electrospinning and biomimetic mineralization, and to study the chemical composition distribution of the coating surface by using the Fourier Transform Infrared microspectroscopy. 

## 2. Materials and Methods

### 2.1. Materials

Common high-purity chemical reagents were supplied from Sigma Aldrich (Milan, Italy). Ultrapure water (0.22 mS, 25°C) was used in all experiments.

### 2.2. Processing of Pure Collagen Scaffolds

The electrospinning apparatus used in this work consists of three components: (i) spinneret, (ii) collector, and (iii) high-voltage power system. The spinneret is directly connected to a syringe, which acts as a reservoir for the collagen solution. Collagen (type I, extracted from equine Achilles tendon using the manufacturing method of Opocrin S.p.A. (Corlo di Formigine, Italy) [21]) was dissolved into CH_3_COOH 0.1 M at the concentration of 2.5 wt%. The solution was fed into a 10 mL glass syringe connected to a stainless steel needle using a Teflon tube having an inner diameter of about 1.0 mm, controlled by a syringe pump (KDS200, KD Scientific Inc., USA), at a feeding rate of 0.01 mL/min. A plate-type titanium alloy (ASTMF136 ELI, Titanium International Group S.r.l., Sala Bolognese, Italy) was used as collector as well as substrate for the electrospun fibers. The distance between the spinneret tip and the collector was adjusted to 18 cm. A voltage of 15 kV was applied across the spinneret and the collector by a voltage-regulated DC power supply (SL150, Spellman High Voltage Electronics, USA) to generate the electrically charged collagen jet. The titanium coated with the electrospun collagen fibers was vacuum dried overnight before carrying out the characterizations.

### 2.3. Processing of Mineralized Collagen Scaffolds

**Figure 1:**
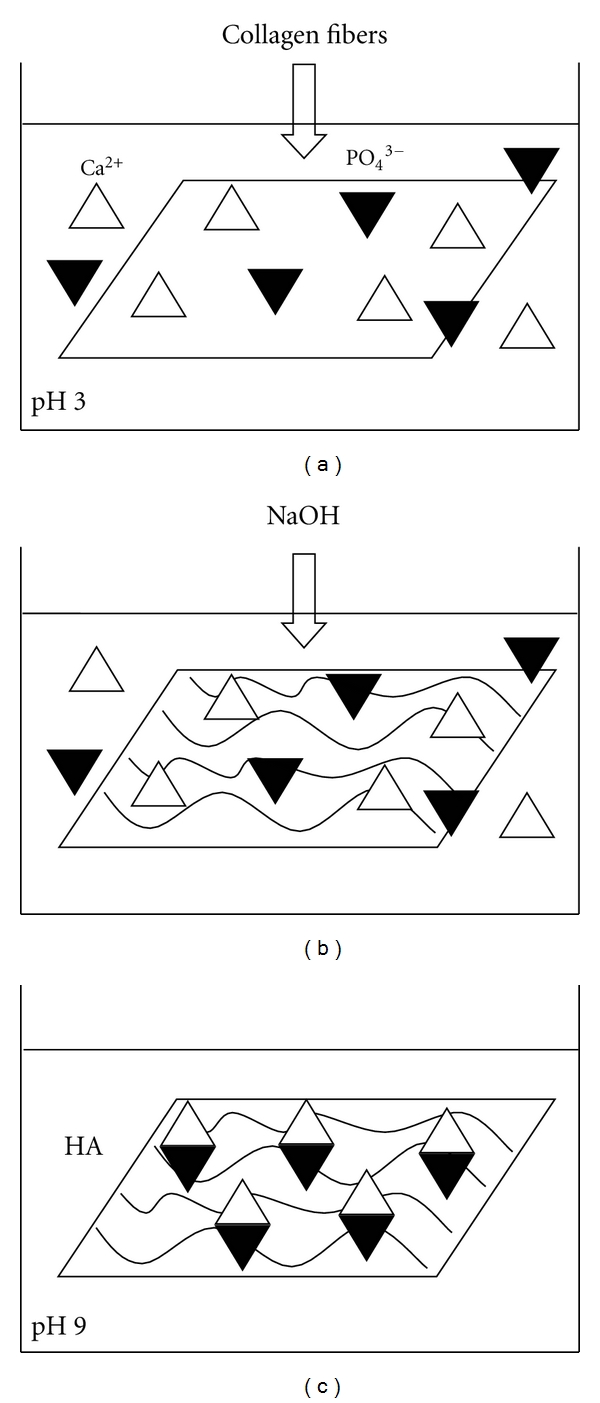
Scheme of the processing steps involved in the electrospun nanostructured collagen-hydroxyapatite fibers preparation: (a) electrospinning of collagen in CH_3_COOH solution into an aqueous calcium and phosphate precursor solutions (Ca(CH_3_COO)_2_ and NH_4_H_2_PO_4_, resp.) at pH 3; (b) hydrolysis with NaOH to pH 9; (c) aging the composite in reaction mixture for 15 min, followed by harvesting and vacuum drying.

The processing steps involved in the preparation of electrospun fibers of collagen-hydroxyapatite are schematically illustrated in Figure 1. The electrospinning of collagen was carried out by the same method described above, but for the preparation of mineralized collagen, the collector was immersed in 10 mL of an aqueous solutions of Ca(CH_3_COO)_2_ 8.5 mM and NH_4_H_2_PO_4_ 5.1 mM at pH 3.0, keeping constant the Ca/P molar ratio of 1.67. At the end of collagen deposition, the reaction mixture was titrated with NaOH solution to pH 9. After aging for 15 min, the titanium coated with composite was repeatedly washed with ultrapure water and harvested by vacuum dried before carrying out the characterizations.

### 2.4. Scaffolds Characterizations

The surface morphology of the electrospun collagen and the collagen-HA scaffolds was examined by using a scanning electron microscope (SEM) (Carl-Zeiss EVO, 40 XVP microscope) using secondary electrons at 25 kV, equipped with an energy-dispersive detector for X-rays (EDAX; Inca 250, Oxford).

The inorganic phase was analyzed by X-ray diffraction (XRD) using a PanAnalytical X'Pert Pro equipped with an X'Celerator detector, using Cu K*α* radiation generated at 40 kV and 40 mA. The instrument was configured with 1/2° divergence and receiving slits. The 2*θ* range was from 20° to 60° with a step size (°2*θ*) of 0.05 and a counting time of 3 s.

Crystal domain size along the HA axis directions were calculated applying the Scherrer equation:

(1) $$L_{\left(\text{hkl}\right)}=\frac{0.94\lambda }{\left[\cos\theta \left(\sqrt{\Delta r^{2}-\Delta_{0}^{2}}\right)\right]},$$

where *θ* is the diffraction angle for (hkl) plane, Δ_*r*_ and Δ_0_ the widths in radians of reflection (hkl) at half height for the synthesized and pure hydroxyapatite (standard reference material, calcium hydroxyapatite, NIST), respectively, and *λ* = 1.5405 Å.

 Thermogravimetric analysis (TGA) were carried out on dried samples removed from the titanium alloy plate using a Thermal Analysis SDT Q 600 (TA Instruments, New Castle, DE, USA). Heating was performed in a nitrogen flow (100 mL min^−1^) using an alumina sample holder. The temperature was increased to 700°C using a heating rate of 10°C/min. The weight of the samples was approximately 3 mg.

Transmission electron microscopy (TEM) investigations were carried out using a CM 100 instrument (80 kV) (Philips, Eindhoven, The Netherlands). The samples were removed from the titanium alloy plate and suspended in ultrapure water. A drop of the coating material suspension was deposited on holey-carbon foils supported on conventional copper microgrids. The samples were observed after staining at room temperature for 30 minutes with 0.5% aqueous uranyl acetate solution.

 Infrared microscopy spectral data were recorded by a Perkin-Elmer Spectrum One Fourier Transform Infrared Spectrometer (FT-IR) equipped with a Perkin-Elmer Autoimage microscope. The spatial resolution was 50 × 50 *μ*m^2^ and the spectral resolution was 4 cm^−1^. The spatial resolution of 50 × 50 *μ*m^2^ was chosen in order to optimize the signal-to-noise ratio and considering the high homogeneity of the samples. The spectra were collected in reflectance mode on titanium plate and they are related to the surface of the sample. Specific areas of interest were identified by a microscope television camera and for each one, an IR image was acquired using a liquid nitrogen cooled, 16-pixel mercury cadmium telluride (MCT-A) line detector at a 25 *μ*m/pixel resolution. Baseline (polynomial line fit), smoothing, and Amide I normalizations were performed in all cases [22]. The correlation maps allow to evidence the chemical topographic distribution of a selected spectrum on the analyzed area. According to a colorimetric scale, the white colour corresponds to a zone with maximum absorption, while the dark colour refers to a zone in which the corresponding absorption band is absent [22].

### 2.5. Statistic Analysis

All experiments were performed at least three times. Determination of HA crystallite domain size along the the *c*-axis and along the perpendicular to it was carried out 5 times on the same synthesis product.

## 3. Results and Discussion

In order to maintain the fibrous structure of the polymer, electrospun collagen in the form of nanofibrous scaffolds was produced. The spinning conditions to obtain collagen scaffolds were optimized by manipulating the experimental parameters, such as concentration of solution, distance between the spinneret and the collector, and the flow rate, in order to produce fibrous structure without any bead formation. By optimizing so (see Section 2), uniform fibers with three-dimensional pore structure with high porosity, appropriate for self-assembling mineralization, have been formed. These findings are comparable to the results reported by Boland et al. [23] for electrospun type I collagen fibers with average fiber diameters ranging from about 100 nm to 4.6 *μ*m depending on the concentration. In line with previous reports on other polymeric materials [24], we conclude that electrospinning of collagenous proteins at concentrations above 2.5 wt% will yield smooth and uniform fibers of several hundred nanometers in diameters. By contrast, electrospinning at lower concentrations will result in small fibers, but with beads.

**Figure 2:**
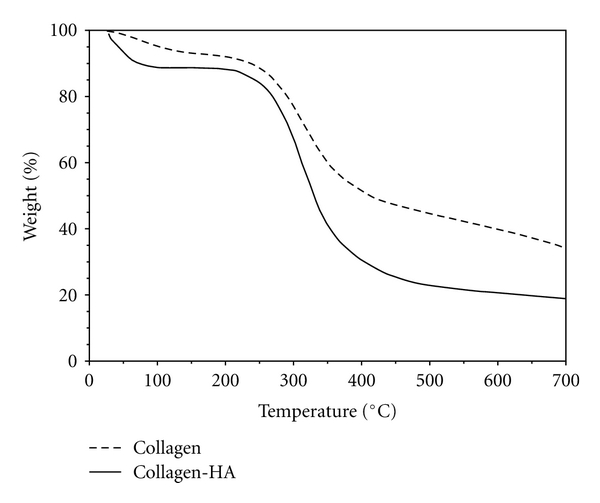
TGA curves of electrospun pure collagen (straight line) and collagen-hydroxyapatite composite (dotted line).

The self-assembly process to nucleate HA crystals depends on the negatively charged carboxyl chemical groups of collagen that can bind Ca^2+^ and on the pH of the reaction medium. The final pH value of 9.0 has been chosen in order to induce the HA crystallization in the optimal condition [25]. In order to optimize the HA crystallization, different experiments have been carried out using several calcium and phosphate salts at different concentrations by keeping the Ca/P ratio constant and equal to the HA stoichiometric value of 1.67. The use of Ca(CH_3_COO)_2_ 8.5 mM and NH_4_H_2_PO_4_ 5.1 mM was found to be the optimal composition to precipitate HA crystals. Furthermore, these values have been chosen in order to preserve the fiber-like morphology, in fact, at the higher HA amounts, a considerable level of beads was formed instead of the development of a fibrous morphology [26]. It is reasonable that when the HA amount was high, the collagen could not effectively distribute the HA nanocrystals during the precipitation process due to the relatively low density of its amino acid chains. As a result, some of the HA nanocrystals could be precipitated in large clusters without the direct involvement of the amino acids of collagen. In order to quantify the amount of apatite in the mineralized collagen, TGA investigations were carried out (Figure 2). TGA curves of pure collagen and mineralized collagen showed similar profile with loss in the range from room temperature to 200°C due to the evaporation of physisorbed water and weight loss between 200 and 500°C associated with the decomposition of collagen molecules followed by a slight loss between 500 and 700°C resulting from the combustion of the residual organic components [27]. Considering the residue of pure collagen (~17 wt%), the apatitic content in the mineralized composite was determined as about 20 wt%.

**Figure 3:**
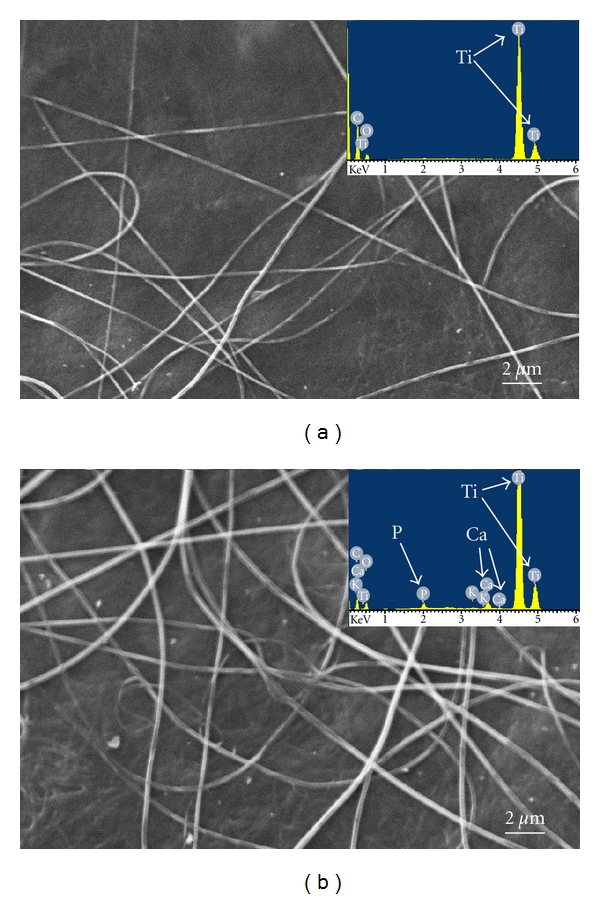
Scanning electron microscopy (SEM) images and energy dispersive X-rays (EDAX) analysis of electrospun pure collagen (a) and collagen-hydroxyapatite composite (b).

 The morphology of the electrospun material was studied by SEM, and the micrographs of the pure collagen and mineralized collagen scaffolds are shown in Figure 3. The electrospun fibers of pure collagen have randomly oriented features trough out the matrix, as well as the mineralized fibers. The average diameters of polymers fibers formed during electrospinning with and without HA, determined by SEM, were about 200 nm. These values are well comparable with ECM fibers, which are in the range of 50–500 nm [2]. Finally, the two materials appear very similar in terms of morphology and dimensions of the fibers, and the visualization of the HA crystals was not allowed by SEM probably due to their very small dimensions in the range of 20–30 nm [28].

 The presence of deposited HA is evidenced by the EDAX spectrum collected for the mineralized sample (inset in Figure 3(b)). This spectrum indicates that the intensity ratio of Ca and P signals was about 1.5 coherently with the value of biological HA [3].

**Figure 4:**
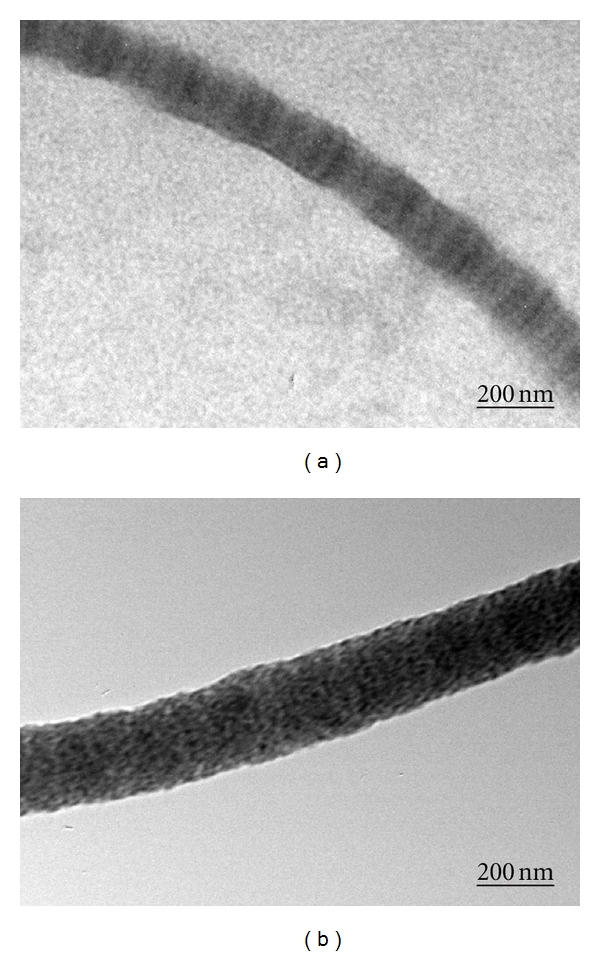
Transmission electron microscopy (TEM) images of electrospun pure collagen (a) and collagen-hydroxyapatite composite (b).

TEM images of the reconstituted electrospun collagen and collagen-hydroxyapatite are shown in Figures 4(a) and 4(b), respectively. The average diameters of polymers fibers were very similar (about 200 nm) in agreement with the SEM observations. The stained pure collagen fibers display a high degree of fibril assembly, evidencing the characteristic D-band pattern with a regular period of 67 nm along the long axis of the fibril [29]. On the contrary, the mineralized fibers do not show the D-band pattern due to the presence of HA.  Figure 4(b) clearly illustrates the presence of HA nanocrystals of about 20–30 nm in size that completely cover the surface of collagen fibers.

**Figure 5:**
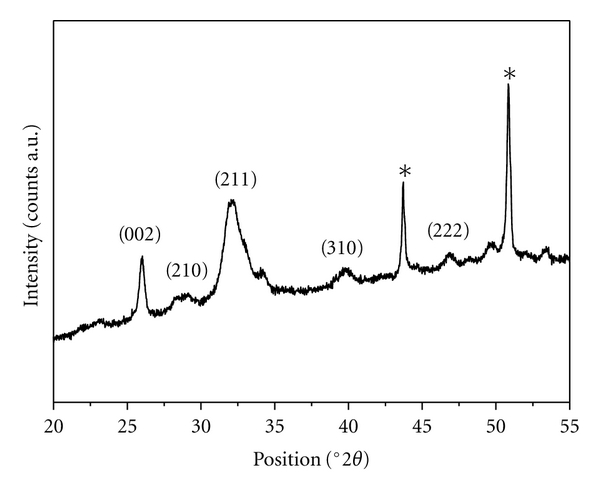
X-Ray diffraction (XRD) pattern of collagen-hydroxyapatite composite; *indicates the Ti alloy diffraction peaks.

 To clearly identify the mineral phase developed during the mineralization of the electrospun collagen fibers, the XRD was employed and the pattern is shown in Figure 5. All of the mineral diffraction peaks were indexed to HA pure phase (JCPDS file number 9-432) and no other impurity phases were detected. The most intense peaks at 43.7° and 50.8° 2*θ* are due to the titanium alloy substrate (marked with * in Figure 5) [30]. The diffraction pattern of the HA exhibits not well-defined diffraction maxima, in fact the peaks corresponding to the crystallographic planes (211), (121), and (300) were all combined into one broad peak centred at about 32° 2*θ*, indicating a relatively low degree of crystallinity and nanosized dimensions [4]. The crystal domain sizes along the *c*-axis (D_002_) and along the perpendicular to it (D_310_), using the 2*θ* = 26° (002) and 2*θ* = 39° (310) diffraction peaks, respectively, were 25 ± 5 nm and 11 ± 3 nm. These values are in agreement with the TEM observations. It is worth to notice that the HA diffraction pattern was very similar to that recorded for the deproteinated bone apatite and this was also confirmed by the similarity of the crystal domain size along the *c*-axis (21 nm) [3].

**Figure 6:**
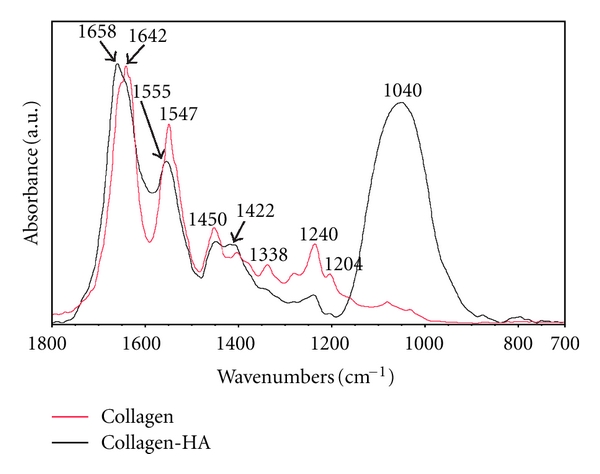
FT-IR spectra of electrospun pure collagen (red) and collagen-hydroxyapatite composite (black) in the region 1800–700 cm^−1^.

 The FT-IR spectra of electrospun pure collagen and mineralized collagen are depicted in Figure 6. The spectrum of electrospun collagen shows all the characteristic bands of collagen at 1204, 1240, 1280, and 1338 cm^−1^ arising from C–OH stretching modes and 1400 and 1450 cm^−1^ associated with the asymmetric and symmetric CH_3_ bending vibrations [4]. The absorbance ratio of the bands 1240/1450 cm^−1^ which is a measure of the integrity of the triple-helix structure [31] was about 1.0 suggesting that collagen triple helix secondary structure was conserved. The spectra of collagen-HA composite displays the bands of poorly crystalline carbonate-HA, a convoluted band centred at 1040 cm^−1^ (asymmetric stretching modes of the phosphate groups) and a band at 1422 cm^−1^ (antisymmetric stretching mode of C–O, consistent with a carbonate type-B-substituted apatite, where the carbonate ions replace the phosphate ions in the crystal lattice) [4], while the bands of collagen change their positions and their relative intensities. This finding could indicate that during the mineralization procedure, the collagen secondary structure has been modified by the effective interaction with HA nanocrystals. The limited amount of carbonate in the HA derived from CO_2_ dissolved in the preparation media and adsorbed on the surface materials during the previous storage. The presence of carbonate in the structure of apatite was intentionally retained, in order to better mimic the biological ones [3]. In a previous FT-IR study comparing “bulk” collagen with electrospun collagen scaffolds, Stanishevsky et al. [32] reported a shift to lower wavenumbers in electrospun collagen of the major peak in the amide I band from 1667 cm^−1^ (fibers) to 1642 cm^−1^ (bulk), as well as the peak of amide II band from 1574 cm^−1^ (fibers) to 1544 cm^−1^ (bulk). Similar amide I and amide II peak shifts for our pure collagen scaffolds were observed (the wavelengths of the bands related to amide I and amide II are 1642 and 1547 cm^−1^, resp.). It was suggested that changes in the triple-helix structure of collagen fibrils during the fiber drawing in the electrified jet may account for this infrared shift. Other changes in the infrared spectra were reported (such as a blue shift of the amide I band) by the addition of apatite nanoparticles to the electrospun collagen fibers [32]. Similar peak shifts in the amide I and amide II of collagen-apatite scaffolds were clearly observed (1642 to 1658 cm^−1^ and 1547 to 1555 cm^−1^, resp.). The observed changes in the infrared spectra are associated with the chemical interaction between the apatite nanoparticles and collagen, in particular they are due to the chemical link between the collagen carboxyl groups and the calcium of HA.

 FT-IR microspectroscopy was used to analyze the electrospun coatings with the aim to map and characterize the chemical distribution of the sample. FT-IR microspectroscopy allows to define the structural interactions between HA nanocrystals and reconstituted collagen fibers. Moreover, this technique is able to verify the topographic and quantitative distribution of the components in the sample.

**Figure 7:**
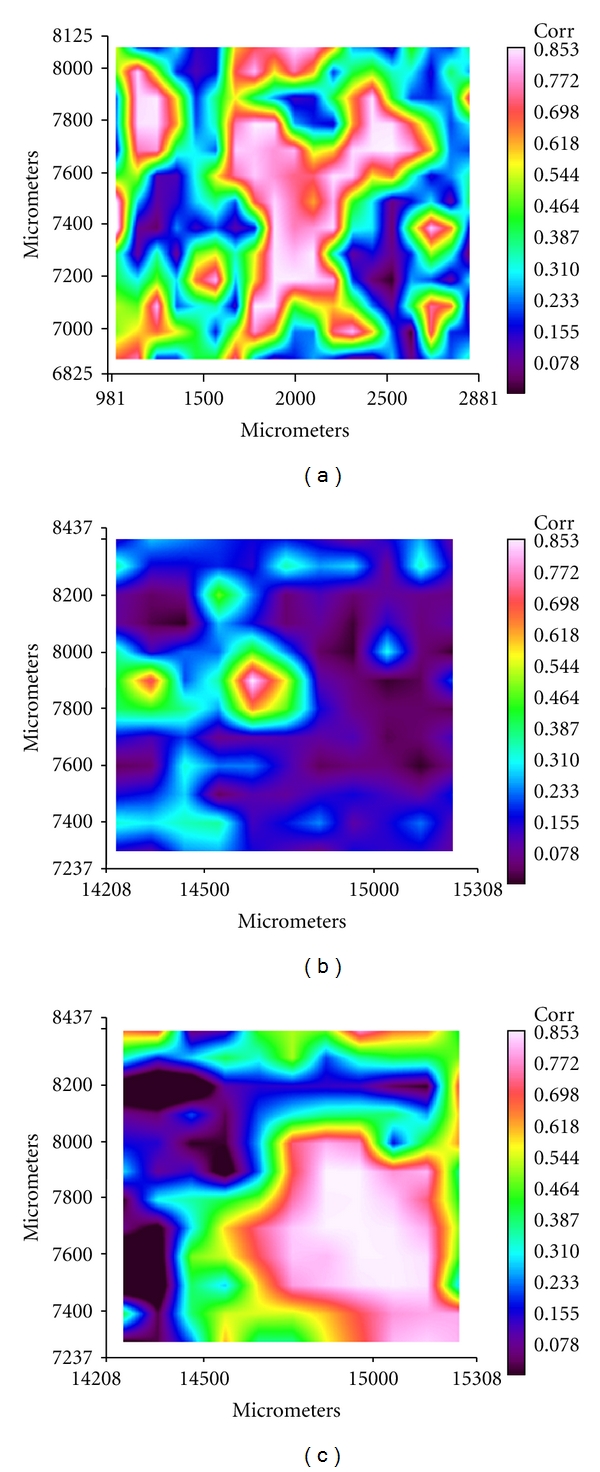
Correlation maps obtained by loading the representative FT-IR spectrum of collagen (Figure 6, red spectrum) in the chemical maps of electrospun pure collagen (a) and mineralized collagen (b) and correlation map obtained by loading the FT-IR spectrum of collagen-hydroxyapatite composite (Figure 6, black spectrum) in the chemical map of mineralized collagen (c).

In Figures 7(a) and 7(b), the correlation maps obtained by loading the representative spectrum of collagen (Figure 6, red spectra) in the chemical maps of electrospun pure collagen (1300 × 1900 *μ*m) and mineralized collagen (1200 × 1100 *μ*m) samples, respectively, are shown. The predominance of a dark area in the Figure 7(b) is related to the absence of the pure collagen, due to the mineralization of all the collagen fibers. On the contrary, Figure 7(c) shows the correlation map obtained by loading the spectrum of collagen-HA (Figure 6, black spectra) in the same chemical map of mineralized collagen sample (1200 × 1100 *μ*m) represented in Figure 7(b). In this case, the representative bands of HA prevail on those of pure collagen and the large white area allows to visualize the crystallization of HA over the protein.

 It is well accepted that the formation of HA on the surfaces of electrospun fibers was controlled by the charged density of chemical groups on the fiber surface. Previous investigations about the nucleation sites of HA crystals on collagen fibers have suggested that the binding of calcium ions on the negatively charged carboxylate groups of collagen is one of the key factors for the first-step nucleation of HA crystals [33]. Collagen has large number of negatively charged carboxyl chemical groups that can bind Ca^2+^ ions to nucleate the HA crystal growth. The carboxyl groups are present in about 11% of the amino acid residues of collagen molecules. Moreover, in a neutral solution, more than 99% of the carboxyl groups of aspartyl and glutamyl ionize favouring the chelation of calcium ions. The carboxyl groups on the outside of the collagen threefold spiral are one kind of site for collagen mineralization [34]. In this way, higher content and smaller crystal size of HA could be formed on fibers with higher densities of carboxyl groups. Carboxyl groups were initially combined with calcium ions through electrostatic attraction, and then phosphoric ions, which were considered to promote the nucleation of HA, until the whole coverage of the fiber with the primary layer of HA was completed. This hypothesis is well supported in this work by the FTIR and TEM investigations and, in particular, by the blue shift of the amide I and amide II bands in the spectra of mineralized collagen. Moreover, by using FT-IR microspectroscopy, we found that part of the HA crystallized outer the fibers, coherently with the fact that the electrospinning technique allows the formation of the collagen fibers and in this case, the carboxyl groups outside the fibers are responsible for the HA nucleation.

## 4. Conclusion

In this paper, a new method to produce collagen-HA composites on titanium alloy substrate, by using electrospinning and biomimetic mineralization techniques, has been developed. The results show that the mineralized scaffolds have the most desirable morphological, structural, and chemical compositional features similar to natural bone ECM, which is a good result of the efficacy of the combinational method of this study. The topographic distribution of the coating components has been evaluated by using the FT-IR microspectroscopy, highlighting the fact that with this method the scaffold is composed by collagen fibers, assembled by the electrospinning, covered by poorly crystalline HA nanosized crystals (20–30 nm in size). The spectroscopic investigation has also suggested that the surface functional groups of the collagen-based material, such as carboxyl groups, are crucial for the mineralization* in vitro*. These fibrous nano-composites should have potential applications as coating materials to improve the biological performances of the titanium medical devices, as scaffolds for tissue engineering, and as fillers for fiber-enforced composites with the aim to improve the bone implant contact and the interaction with the biological environment.
